# Conformational Behavior, Topographical Features, and Antioxidant Activity of Partly De-Esterified Arabinoxylans

**DOI:** 10.3390/polym13162794

**Published:** 2021-08-20

**Authors:** Yubia De Anda-Flores, Elizabeth Carvajal-Millan, Jaime Lizardi-Mendoza, Agustin Rascon-Chu, Judith Tanori-Cordova, Ana Luisa Martínez-López, Alexel J. Burgara-Estrella, Martin R. Pedroza-Montero

**Affiliations:** 1 Biopolymers-CTAOA, Research Center for Food and Development (CIAD, A.C.), Carretera Gustavo Enrique Astiazarán Rosas No. 46, Hermosillo 83304, Mexico; yubia.deanda@estudiantes.ciad.mx (Y.D.A.-F.); jalim@ciad.mx (J.L.-M.); 2 Biotechnology-CTAOV, Research Center for Food and Development (CIAD, A.C.), Carretera Gustavo Enrique Astiazarán Rosas No. 46, Hermosillo 83304, Mexico; arascon@ciad.mx; 3 Department of Polymers and Materials Research, University of Sonora, Hermosillo 83000, Mexico; jtanori@unison.mx; 4 NANO-VAC Research Group, Department of Chemistry and Pharmaceutical Technology, University of Navarra, 31008 Pamplona, Spain; amlopez@unav.es; 5 Department of Physics Research, University of Sonora, Hermosillo 83000, Mexico; alexel.burgara@unison.mx (A.J.B.-E.); martin.pedroza@unison.mx (M.R.P.-M.)

**Keywords:** arabinoxylan, ferulic acid, partial de-esterification, macromolecular characteristics

## Abstract

This study aimed to investigate the effect of arabinoxylans (AX) partial de-esterification with feruloyl esterase on the polysaccharide conformational behavior, topographical features, and antioxidant activity. After enzyme treatment, the ferulic acid (FA) content in AX was reduced from 7.30 to 5.48 µg FA/mg polysaccharide, and the molecule registered a small reduction in radius of gyration (RG), hydrodynamic radius (Rh), characteristic ratio (C∞), and persistence length (q). A slight decrease in α and a small increase in K constants in the Mark–Houwink–Sakurada equation for partially de-esterified AX (FAX) suggested a reduction in molecule structural rigidity and a more expanded coil conformation, respectively, in relation to AX. Fourier transform infrared spectroscopy spectra of AX and FAX presented a pattern characteristic for this polysaccharide. Atomic force microscopy topographic analysis of FAX showed a more regular surface without larger hollows in relation to AX. The antioxidant activity of FAX, compared to AX, was reduced by 30 and 41% using both 2,2′-azino-bis (3-ethylbenzothiazoline-6-sulphonic acid) (ABTS^+^) and 1,1-diphenyl-2-picryl-hydrazyl (DPPH) methods, respectively. These results suggest that feruloyl esterase treatment of AX could offer a strategy to tailor AX chains conformation, morphological features, and antioxidant activity, impacting the development of advanced biomaterials for biomedical and pharmaceutical applications.

## 1. Introduction

Arabinoxylans (AX) are non-starch polysaccharides that mainly form the cell wall of cereal grains. AX can also be recovered through chemical or enzymatic treatments from cereal by-products such as Distiller’s dried grains with solubles (DDGS), the principal maize by-product from the ethanol industry [1]. In the cell wall of grains, AX are cross-linked with other components like cellulose microfibrils by hydrogen bonds, which confers specific stability characteristics [2,3]. AX polymeric chain consists of xylose in β-1,4 with ramifications of α-L-arabinofuranose in α-1,3 and α-1,2. Arabinose can be esterified with ferulic acid (FA). Small amounts of dimers (di-FA) and trimers (tri-FA) of FA can also be present in AX [4,5,6]. The 8-*O*-4′, 8-5′, 5-5′, and 8-8′ di-FA isomers have been detected in AX [7]. The FA content in AX from different by-products such as wheat bran, maize waste-water, and DDGS has been previously investigated [6,8,9]. In AX from nixtamalized maize bran, FA, di-FA, and tri-FA content of 0.54, 0.77, and 0.39 µg/mg polysaccharide, respectively, has been reported [10] while AX from DDGS have been found to carry higher FA (7.53 µg/mg) but lower di-FA and tri-FA (0.53 and 0.04 µg/mg) contents [8]. Previous studies have shown that AX exhibit antioxidant activity, which has been associated with their FA content [11,12]. The antioxidant activity is related to phenolic acids because they can scavenge free radicals and prevent the oxidation of biological substances important to human health [13]. On the other hand, enzymatic modification of polysaccharides such as AX can change their molecular characteristics and functionality [14]. Particularly, feruloyl esterase (EC 3.1.1.73) can hydrolyze ester bonds of FA-arabinose linkages in AX [15,16]. The use of this enzyme to decrease the FA content in AX has been previously studied [12]. Nevertheless, the investigation used AX moderately ferulated (3.27 µg FA/mg polysaccharide), and the resulting products were similar. It has been suggested that AX macromolecular characteristics, such as molecular weight, intrinsic viscosity, arabinose/xylose ratio, and FA content, define the polysaccharide functional properties [17,18]. However, chain conformation and, particularly, parameters related to chain flexibility, such as the chain persistence length (q) and the Mark–Houwink–Sakurada exponent α have not been previously studied in AX partially de-esterified with feruloyl esterase. Besides, to the best of our knowledge, atomic force microscopy analysis of feruloyl esterase treated AX has not been reported elsewhere. In this regard, it is important to investigate the effect of enzymatic partial de-esterification of AX presenting a high FA content on the macromolecule conformation, topography, and functionality. This work aimed to study the effect of partial de-esterification with feruloyl esterase of AX on the polysaccharide conformational behavior, topographical features, and antioxidant activity. This approach could be an opportunity to tailor AX with different FA content, which could be used to design advanced biomaterials with potential applications in the biomedical and pharmaceutical industry.

## 2. Materials and Methods

### 2.1. Materials and Reagents

AX were extracted from DDGS, as recently reported [19]. Laccase (E.C.1.10.3.2) from *Trametes versicolor* and the chemicals used were purchased from Sigma Aldrich Co. (St. Louis, MO, USA). Feruloyl esterase (FAE) (E.C.3.1.1.73) was kindly provided from Biocatalysts Limited (Cardiff, UK) (product number PDN N1/11).

### 2.2. Partial De-Esterification of AX by Feruloyl Esterase

AX dispersion (2% *w*/*v*) was prepared in MOPS buffer (100 mM, pH 6) stirred at 25 °C for 12 h. Feruloyl esterase was added to AX dispersion (721 µU enzyme/mg polysaccharide) at 40 °C in the dark over 24 h gentling stirring. The enzymatic reaction was stopped by adding glacial acetic acid (1:5 *v*/*v*). Then, the reaction mixture was allowed to precipitate in ethanol 86% (*v*/*v*) for 12 h at 4 °C. The precipitate was recovered and dried by solvent exchange (80% (*v*/*v*) ethanol, absolute ethanol, and acetone) to give partially de-esterified AX (FAX) [12].

### 2.3. Phenolic Acids Analysis

The quantification of FA, di-FA, and tri-FA in AX and FAX powder were determined by high-performance liquid chromatography (HPLC) (Waters Co, Milford, MA, USA) with photodiode array detector Waters 996 (Millipore Co., Milford, MA, USA) and an Alltima C18 column (250 × 4.6 mm; Alltech Associates, Inc., Deerfield, IL, USA). The samples (50 mg of AX and FAX) were saponified (2 mL of 2 N NaOH) and maintained in agitation at 100 rpm and 35 °C for 2 h in darkness. Then, 100 µL of 3,4,5-trimethoxycinnamic acid (TMCA) and 5 mL of 4 N hydrochloric acid were added to the samples (adjusted pH 2.0). The phenolic acids were extracted twice with 5 mL of diethyl ether and then evaporated to dryness at 40 °C under nitrogen gas (Dri-Block DB-3A, Techne, UK). The extract was recovered in 1 mL of methanol: water (50:50) and filtered (0.45 µm, Millipore). Detection was followed by UV absorbance at 320 nm. Gradient elution was performed using acetonitrile and sodium acetate buffer (0.05 M, pH 4.0) at 1 mL/min at 35 °C, in linear gradients from 15/85 to 35/65 in 30 min, 35/65 to 60/40 in 0.5 min, 60/40 to 15/85 in 4.5 min, and finally maintained at 15/85 for 5 min [20,21].

### 2.4. Fourier Transform Infra-Red (FTIR) Spectroscopy

FTIR spectra of dry AX and FAX powder were recorded on a Nicolet iS50 FT-RI Spectrometer (Madison, WI, USA). The samples were examined by iS50 ATR analysis. Spectra were recorded in from 4000 to 400 cm^−1^ range [22].

### 2.5. Macromolecular Characteristics

Weight-average molar mass (*M*w), number-average molar mass (Mn), intrinsic viscosity ([*η*]), radius of gyration (RG), hydrodynamic radius (Rh), and polydispersity index (I = *M*w/Mn) were determined using a size exclusion chromatography (SEC) system on a DAWN HELOS-II 8 multi-angle laser light scattering (MALS) instrument detector coupled with a ViscoStar-II Viscometer and a refractive index (RI) Optilab T-rex detector (Wyatt Technology Corp., Santa Barbara, CA, USA). Samples were dissolved in 50 mM NaNO_3_/0.02% NaN_3_ at 5 mg/mL and 80 °C for one h, then centrifuged (15,000 rpm, 10 min) and filtered (0.45 µm, Millipore). A flow rate of 0.7 mL/min in an Agilent HPLC System was used (G1310B Iso-Pump, G1329B autosampler, and G1314F Variable Wavelength Detector, Agilent Technologies, Inc., Santa Clara, CA, USA). The samples were injected into two columns: Shodex OH-pak SBH-Q-804 and 805 (Shodex Showa Denco K.K., Tokyo, Japan). The ASTRA 6.1 software was used. The specific refractive index increment (*dn/dc*) value of 0.146 mL/g was used [23]. The characteristic ratio (C∞) and persistence length (q) were calculated as previously described [23] using the following equations:

q = (C∞+1) × I_0_/2 (1)

where C∞ is the characteristic ratio

C∞ = 6·RG^2^ × M_0_/I_0_^2^ × *M*w (2)

where I_0_ = 0.54 nm (length of a β-d-xylopyranose residue), M_0_ = 132 g/mol (molar mass of an anhydro-xylose residue), and *M*w the molar mass of the xylan backbone.

### 2.6. Atomic Force Microscopy (AFM)

AFM was used to characterize the topographical features of AX and FAX dispersions [7]. Polysaccharide dispersions were prepared by dissolving 5 µg/mL of sample in 50 mM sodium acetate buffer at pH 5.5. One drop of each dispersion was immediately deposited onto the mica surface and allowed to dry in Petri dishes for one h at 25 °C [24]. AX and FAX film images were obtained using a microscope model Alpha3000 RA (WITec, Germany) using a 20× objective combined with an 8 nm tipped measuring needle. The probe had a spring constant of 42 N/m and a resonant frequency of 285 kHz. The analysis of five membrane fragments (5 × 5 µm) per specimen was obtained. Images and their profiles were analyzed in 3D models with the software program WITec project FOUR v4.1.

### 2.7. Antioxidant Activity

The antioxidant activity of AX and FAX powder was measured using the 2,2′-azino-bis (3-ethylbenzothiazoline-6-sulphonic acid) (ABTS^+^) and 1,1-diphenyl-2-picryl-hydrazyl (DPPH) assays.

#### 2.7.1. ABTS^+^

The ABTS^+^ assay was performed as previously described [12,25,26]. The ABTS^+^ reagent was prepared by mixing ABTS^+^ (34 mg) at a final concentration of 7 mM with 10 mL of 2.45 mM potassium persulfate, keeping at room temperature for ~16 h. The ABTS^+^ was mixed with ethanol: water (50/50 *v*/*v*) (Abs_734_ of 0.7 ± 0.02). Each sample (1 mg of AX and FAX) was mixed with 3 mL of the ABTS^+^ reagent for 2 min and finally centrifugated at 9200× *g* for 2 min. Absorbance at 734 nm was measured (7, 15, and 21 min) for the ABTS^+^ reagent mixture. After mixing the samples with the ABTS^+^ reagent, the supernatant was measured at 14 and 21 min. The antioxidant activity was expressed as µmol of Trolox (6-hydroxy-2,5,7,8-tetramethylchoman-2-carboxylic acid) equivalent antioxidant activity per gram of sample (µmol TEAC/g). A dose–response curve for Trolox was performed at different concentrations (0–150 µg/mL).

#### 2.7.2. DPPH

The DPPH assay was performed as previously described [12,27] with some modifications. The dose–response curve of Trolox was performed at different concentrations (0.095–18.75 µg/mL). The absorbance of the medium sample supernatant was measured at 515 nm at 40 y 60 min. DPPH work solution (1.8 mg; final concentration 91.3 µM) was dissolved in 50 mL of methanol: water (60:40) and stored in the dark. The sample dissolution volume was mixing 400 µL (1 mg AX/mL water), 250 µL of methanol, and 750 µL of DPPH (final concentration 45 µM) and kept in the dark for 35 min. The results were expressed as µmol TEAC/g sample.

### 2.8. Statistical Analysis

Chemical analyses were carried out by triplicate, and results were expressed as mean ± SD. Statistical analysis was performed using Mann–Whitney U-test, *p* < 0.05 was considered statistically significant.

## 3. Results and Discussion

### 3.1. AX and FAX Phenolic Acids and Macromolecular Characteristics

The FA content in AX (7.30 µg/mg polysaccharide) was in the range reported in the literature for other maize AX [7,8]. The esterified FA in the AX chain depends on the conditions of the method used to extract it from diverse types of plants, cereals, or agro-industrial by-products [8,18,22,28]. After enzymatic treatment, partially de-esterified AX (FAX) presented a FA content of 5.48 µg/mg, which corresponds to a 25% FA content decrease in the sample (Table 1). AX and FAX contained small amounts of di-FA structures (Table 1). The comparative percentages were 43, 48, and 9% in AX and 39, 46, and 14% in FAX for 8-5′, 5-5′ and 8-*O*-4′ di-FA isomers, respectively. The tri-FA structure was not detected in these samples. The di-FA content was not affected after de-esterification treatment, proving that the enzyme only acts on the FA residues. The 8-5′ and 5-5′ di-FA structures have been previously identified as the most abundant isomers in AX from DDGS [12]. The 8-O-4′ di-FA presented a significant increase after the de-esterification treatment.

**Figure 1:**
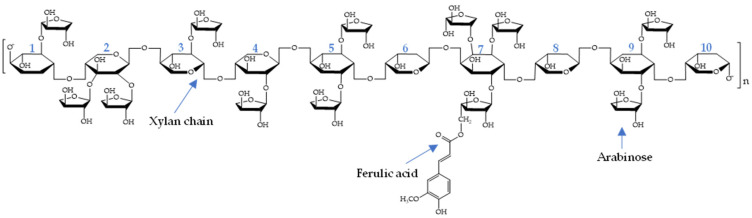
Schematic representations of FA substituent at the AX chain.

The arabinose to xylose ratio (A/X) for AX and FAX was 1.16 and 1.15, respectively (Table 2), indicating a highly branched structure. The A/X values reported for maize bran AX range from 0.72 to 1.10, corresponding to moderate or highly branched structure [8,10,29]. A previous study [12] reported a 61.5% FA content reduction (from 3.27 to 1.26 µg FA/mg sample) in AX presenting an A/X = 0.69 using this enzyme. In the present investigation, AX showed a higher initial FA content (7.30 µg/mg polysaccharide) and A/X ratio (1.16) than the values reported by those authors. The structural complexity of the AX used in this work (higher FA content and arabinose substitution) could explain the small amount of FA released by feruloyl esterase as it could reduce the feruloyl esterase access to the FA-arabinose ester link. In the present study, the FA content in AX and FAX corresponded to approximately 1 FA substitution per 95 and 126 arabinose residues, respectively, close to the value previously reported for wheat AX treated with a feruloyl esterase (1 FA substitution per 120 arabinose residues) [30]. In addition, in the current investigation, AX and FAX showed 1 FA substituent per 82 and 110 xylose residues, respectively. In contrast, wheat AX shows a lower substitution of FA (220 xylose residues in the AX backbone per FA monomer) [30], which confirms a higher degree of ferulation in maize AX than in wheat AX (Figure 1).

The macromolecular characteristics of AX and FAX are presented in Table 2. In general, A/X ratio, molecular weight (*M*w), polydispersity index (PI), intrinsic viscosity [*η*], radius of gyration (RG), and hydrodynamic radius (Rh) values were in the range reported for other maize AX [8,31,32]. Characteristic ratio (C∞) and persistence length (q) for AX and FAX were close to the values reported in previous studies for AX extracted from wheat [23,33]. FAX *M*w showed a slight decrease compared to AX, which could be attributed to conformational changes in the molecule after partial FA remotion by feruloyl esterase. A previous study reported a reduction in wheat AX *M*w after polysaccharide FA de-esterification [23]. Concerning [*η*], FAX registered a slight increase regarding AX. The partial FA remotion from the arabinose residues may increase the polysaccharide solubility parameter. A previous study indicates that polymer intrinsic viscosity tends to augment when the solubility parameter of the medium increase [34].

The polysaccharide changes in *M*w and [*η*] after feruloyl esterase treatment could be associated with the different FA content in the polymer. The estimated FA monomer presence through the polysaccharide chain changed from one FA per 95 arabinoses and 82 xyloses in AX to one FA per 126 arabinoses and 110 xyloses in FAX. These differences probably modify the molecule spatial arrangement, resulting in slightly reduced RG, Rh, C∞, and q values in FAX concerning AX. A previous investigation reported C∞ and q values of 15.6 and 4.2 for wheat AX presenting an A/X ratio of 1.3, close to the values registered in the present study [33]. Similar C∞ and q values have been related to a semi-flexible random-coil conformation in AX [23]. FAX presented lower C∞ and q values than AX, suggesting that partial remotion of FA in the polysaccharide reduces the chain rigidity. The Mark–Houwink–Sakurada equation is related to intrinsic viscosity; in this equation, K and α are used to study polysaccharides conformation. The exponent α is related to chain conformation; values of 1.26 and 0.50 correspond to very rigid and random coil structures, respectively. In addition, high K values indicate an expanded coil conformation, while low K values represent a compact coil conformation [35]. In the present study, AX and FAX presented α and K values that suggest a molecular random coil structure, which agrees with the behavior reported for wheat AX [23]. Besides, FAX registered a small decrease in α and a slight increase in K concerning AX, which suggests a reduction in the molecule structural rigidity and more expanded coil conformation, respectively.

**Figure 2:**
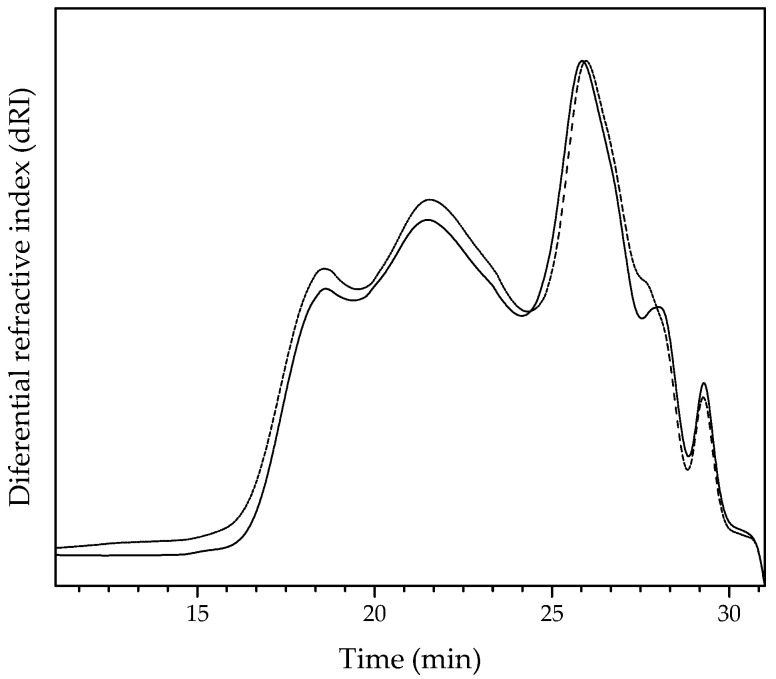
SE-HPLC chromatograms of AX (−) and FAX (--).

The SE-HPLC profile of FAX was similar to the AX pattern, demonstrating that the polysaccharide backbone was not depolymerized by feruloyl esterase treatment (Figure 2). This result indicates that the feruloyl esterase used in the present study does not cleavage the glycosidic linkages in the xylose backbone, arabinose substitutions, or the ester bonds in di-FA and tri-FA that may interconnect the AX chains [30], confirming that this enzyme did not possess xylanase or arabinofuranosidase activity [12]. Overall, the FAX profile appears to be slightly moved to the right from AX, suggesting a minor decrease in the polysaccharide *M*w after enzyme treatment, as reported in Table 2. The slight difference in the SE-HPLC profile could be attributed to changes in the FAX structural flexibility due to the partial FA remotion.

### 3.2. Fourier Transform Infra-Red (FTIR) Spectroscopy

**Figure 3:**
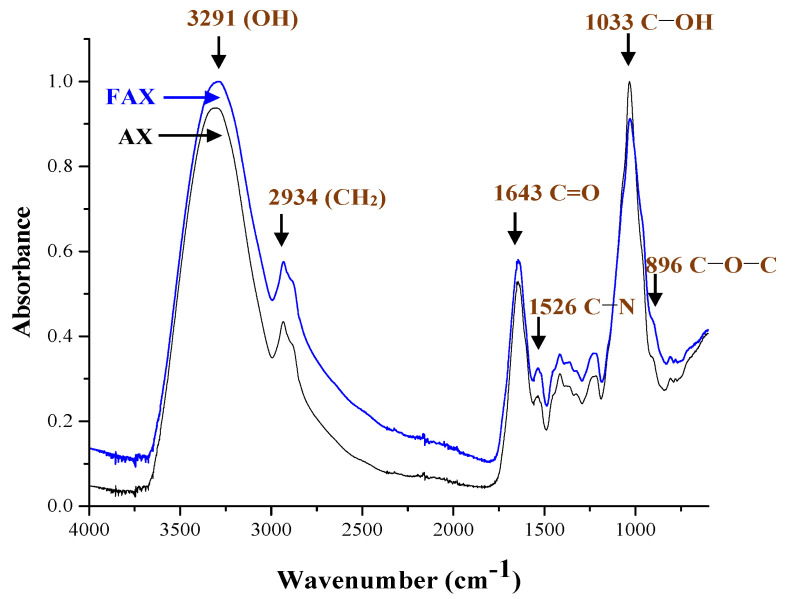
FTIR spectra of AX and FAX.

The FTIR spectra of AX and FAX are presented in Figure 3. Both samples showed a similar pattern in their chemical structure, and the feruloyl esterase treatment did not alter their molecular identity. The characteristic absorption bands at 1200–800cm^−1^ represent the specific region of the polysaccharides [36,37]. The main band is observed at ~1033 cm^−1^, which is assigned to the C−OH bending vibration. The small shoulder at 897 cm^−1^ can be related to the antisymmetric C−O−C stretch mode of the glycosidic link and β (1→4) linkages between the sugar units [22,37]. The phenolic acids and proteins have specific absorption bands in the 1500–1800 cm^−1^, and the bands 1650 and 1533 cm^−1^ are related to amide I and amide II bands, respectively [38,39]. The phenolic acid bands, such as FA, are divided primarily in an absorption band representing a strong aromatic ring vibration at 1517 cm^−1^ and secondary absorption bands (1690, 1620, and 1600 cm^−1^). In the present study, these bands were not clearly defined because of the overlap by the amide I band (1650 cm^−1^) and the amide II band (1533 cm^−1^), which are related to the presence of residual protein attached to the AX chain [12,40]. The band at 3292 cm^−1^ corresponds to the stretching of the OH groups and the band at 2935 cm^−1^ to the CH_2_ groups [8,41,42].

### 3.3. Atomic Force Microscopy (AFM)

**Figure 4:**
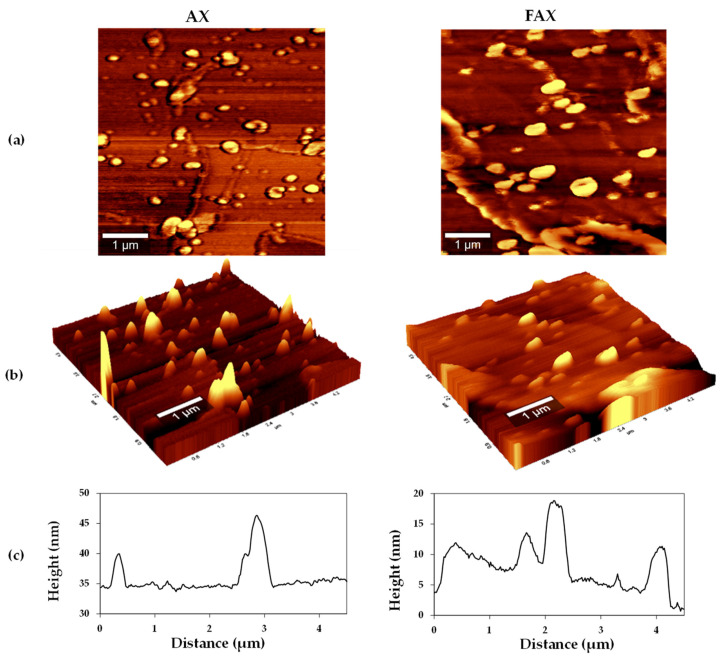
Atomic force microscopy topography analysis of AX (left) and FAX (right) (**a**) 2D surface image (**b**) 3D surface image and (**c**) profile line. Dispersions (5 µm × 5 µm).

The topography of AX and FAX was investigated using AFM (Figure 4). The analysis revealed that the surface morphology slightly changed because of the FA content reduction. It was observed that AX showed a grained and irregular surface, while FAX presented a more regular surface without larger hollows. Root mean square roughness (Rq) values were ~46 nm and ~19 nm for the total surface of AX and FAX, respectively, which confirms previous observations.

The smoother surface registered in FAX about AX may be caused by the superposition of polysaccharide chains presenting additional structural flexibility, as observed in SE-HPLC analysis, facilitating the FAX chains partial aggregation. It has been previously reported that AX molecules analyzed by AFM register an average height of 0.4 or 1.2 nm [43,44,45]. According to those values, AX ad FAX dispersions investigated in the present study could be formed of connected strands presenting 115-38 and 48-16 polysaccharide chains, respectively. Some authors [46,47] reported that the FA spatial position in the AX molecules might vary because the polysaccharide chain is flexible and presents a three-fold helical conformation, which could conduce to variations in their macromolecular arrangement.

### 3.4. Antioxidant Activity

Table 3 shows the AX and FAX antioxidant activity using the DPPH and ABTS^+^ methods based on a mechanism that implies electron transference from the antioxidant agent to the DDPH or ABTS^+^ radicals, respectively [48]. The results show that FAX antioxidant activity decreased by 30 and 41% using the ABTS^+^ and DPPH methods, respectively, about AX. This reduction in the polysaccharide antioxidant activity could be attributed to the remotion of 25% of the FA content in AX. Similar behavior has been previously reported in other partly deferulated AX [49]. The ABTS^+^ values were higher than DPPH values for AX and FAX samples. This difference may result from the higher reactivity of the ABTS^+^ radical concerning DPPH radicals [48]. In a previous study, the antioxidant activity of AX presenting a FA content of 5.45 µg/mg polysaccharide, registered antioxidant activity values of 68.05 and 32.23 µmol TEAC/g by using ABTS^+^ and DPPH methods, respectively [11]. Those values are in the range reported in the present work (Table 3). Another investigation reported antioxidant activity values of 67, and 39 µmol TEAC/g and 28 and 18 µmol TEAC/g for AX (3.27 µg FA/mg polysaccharide) and feruloyl esterase treated AX (1.26 µg FA/mg polysaccharide) following the ABTS^+^ and DPPH method, respectively [12]. The antioxidant activity values reported in that study are in the range presented in Table 3, despite the higher FA contents of the samples used in the present study (7.30 and 5.48 µg/mg polysaccharide), suggesting that this functional property could also be related to other molecule characteristics, such as chain conformation and flexibility. As reported in Table 1, feruloyl esterase treatment did not affect the total di-FA content in AX. However, the 8-*O*-4′ di-FA presented a significant increase in FAX in relation to AX. It has been reported that di-FA isomers present different antioxidant activity with 8-5′ and 8-*O*-4′ registering higher values than 5-5′ form [50]. The di-FA isomers composition in FAX may have favored the polysaccharide antioxidant activity.

## 4. Conclusions

The partial de-esterification of highly ferulated AX using a feruloyl esterase reduced 25% of the polysaccharide FA content. This partial remotion of FA results in a different polysaccharide conformational behavior reflected by a small reduction in RG, Rh, C∞, and q values. Furthermore, regarding AX, a minor decrease in α and a slight increase in K constants in the Mark–Houwink–Sakurada equation in FAX, suggests a less rigid molecule structure and a more expanded coil conformation, respectively. Atomic force microscopy analysis reveals essential differences in AX and FAX topographical features, with FAX showing a more regular surface without large hollows compared to AX. These topographical differences could be related to the polysaccharide structural flexibility and arrangement changes after partial de-esterification. Under the conditions used in the present study, feruloyl esterase treatment of AX reduces the polysaccharide antioxidant activity down to 41%. The enzymatical AX partial de-esterification could offer a strategy for tailoring this polysaccharide, which could be helpful in the design of advanced biomaterials with potential application in the biomedical and pharmaceutical industry. In addition, conformational behavior, topographical features, and antioxidant activity changes in FAX may conduce to differences in polysaccharide-cells interactions as they occur, for example, during colonic microbiota fermentation; further research is needed to explore this possibility.

## Figures and Tables

**Table 1:** Composition of AX and FAX phenolic acids.

Sample	FA *	di-FA			Total di-FA
		5-5′	8-*O*-4′ *	8-5′	
AX	7.30 ± 0.18	0.100 ± 0.005	0.021 ± 0.006	0.090 ± 0.002	0.212 ± 0.009
FAX	5.48 ± 0.18	0.104 ± 0.001	0.033 ± 0.001	0.088 ± 0.001	0.224 ± 0.003

Results expressed in µg/mg polysaccharide. Mean value of triplicate determinations ± SD. * Values in each column are statistically different (*p* < 0.05).

**Table 2:** Macromolecular characteristics of AX and FAX.

Sample	AX	FAX
A/X ratio	1.16	1.15
*M*w (kDa)	661	562
PI (*M*w/Mn)	2.4	2.4
[*η*] (mL/g)	149	155
RG (nm)	40	36
Rh (nm)	22.5	21.9
C∞	14.2	13.4
q (nm)	4.1	3.9
Mark–Houwink–Sakurada α	0.536	0.521
Mark–Houwink–Sakurada K	1.394 × 10^−1^	1.872 × 10^−1^

*M*w: weight-average molar mass; Mn: number-average molar mass; PI: polydispersity index; [*η*]: intrinsic viscosity; RG: radius of gyration; Rh: hydrodynamic radius; C∞: characteristic ratio; q: persistence length (calculated based on unbranched arabinoxylans).

**Table 3:** Antioxidant activity of AX and FAX.

Sample	ABTS^+^ (µmol TEAC/g) *	DPPH (µmol TEAC/g) *
AX	51.1 ± 1.7	35.0 ± 2.9
FAX	35.9 ± 3.0	20.7 ± 3.4

All results were obtained from triplicate determinations (mean ± SD). * Values in each column are statistically different (*p* < 0.05).

## Data Availability

Not applicable.
